# Modified Vaccinia Ankara–Vectored Vaccine Expressing Nucleoprotein and Matrix Protein 1 (M1) Activates Mucosal M1-Specific T-Cell Immunity and Tissue-Resident Memory T Cells in Human Nasopharynx-Associated Lymphoid Tissue

**DOI:** 10.1093/infdis/jiz593

**Published:** 2019-11-19

**Authors:** Suttida Puksuriwong, Muhammad S Ahmed, Ravi Sharma, Madhan Krishnan, Sam Leong, Teresa Lambe, Paul S McNamara, Sarah C Gilbert, Qibo Zhang

**Affiliations:** 1 Department of Clinical Infection, Microbiology and Immunology, Institute of Infection and Global Health, University of Liverpool, Liverpool, United Kingdom; 2 ENT Departments, Alder Hey Children’s Hospital, Liverpool, United Kingdom; 3 ENT Departments, Aintree University Hospital, Liverpool, United Kingdom; 4 The Jenner Institute, University of Oxford, Oxford, United Kingdom; 5 Institute of Child Health, Alder Hey Children’s Hospital, Liverpool, United Kingdom

**Keywords:** Influenza, T -ell immunity, vaccine, antigen-specific T cell, tissue-resident memory T cells (TRM), nasopharynx-associated lymphoid tissue, cytotoxic T cell

## Abstract

**Background:**

Increasing evidence supports a critical role of CD8^+^ T-cell immunity against influenza. Activation of mucosal CD8^+^ T cells, particularly tissue-resident memory T (T_RM_) cells recognizing conserved epitopes would mediate rapid and broad protection. Matrix protein 1 (M1) is a well-conserved internal protein.

**Methods:**

We studied the capacity of modified vaccinia Ankara (MVA)–vectored vaccine expressing nucleoprotein (NP) and M1 (MVA-NP+M1) to activate M1-specific CD8^+^ T-cell response, including T_RM_ cells, in nasopharynx-associated lymphoid tissue from children and adults.

**Results:**

After MVA-NP+M1 stimulation, M1 was abundantly expressed in adenotonsillar epithelial cells and B cells. MVA-NP+M1 activated a marked interferon γ–secreting T-cell response to M1 peptides. Using tetramer staining, we showed the vaccine activated a marked increase in M1_58–66_ peptide-specific CD8^+^ T cells in tonsillar mononuclear cells of HLA-matched individuals. We also demonstrated MVA-NP+M1 activated a substantial increase in T_RM_ cells exhibiting effector memory T-cell phenotype. On recall antigen recognition, M1-specific T cells rapidly undergo cytotoxic degranulation, release granzyme B and proinflammatory cytokines, leading to target cell killing.

**Conclusions:**

MVA-NP+M1 elicits a substantial M1-specific T-cell response, including T_RM_ cells, in nasopharynx-associated lymphoid tissue, demonstrating its strong capacity to expand memory T-cell pool exhibiting effector memory T-cell phenotype, therefore offering great potential for rapid and broad protection against influenza reinfection.

## Teaser

Modified vaccinia Ankara–vectored vaccine expressing nucleoprotein and matrix protein 1 (MVA-NP+M1) activates a substantial increase in anti-influenza M1-specific T cells, including fast-reacting tissue-resident memory T cells in human nasopharynx mucosal tissue. It is therefore a promising mucosal vaccine candidate.

Influenza still causes widespread morbidity and mortality, despite the available vaccines. Current influenza vaccines predominantly induce subtype-specific antibodies toward hemagglutinin. Because hemagglutinin continuously mutates, vaccine composition needs to be updated every year, and vaccine efficacy varies considerably depending on how well the vaccine strains match circulating viruses [1]. There is a need for more effective vaccines that confer broad immunity against influenza, including those with potential to cause pandemics.

Although neutralizing hemagglutinin-specific antibodies are considered the major protective responses [2], increasing evidence supports an important role for CD8^+^ T-cell–mediated immunity. In individuals experimentally infected with influenza, virus-specific cytotoxic T-cell killing reduced virus shedding in absence of specific antibodies [3]. Preexisting cytotoxic CD8^+^ T cells were associated with decreased disease severity in patients infected with pandemic H1N1 virus [4].

The majority of influenza virus-specific CD8^+^ T cells recognize epitopes shared among virus subtypes, including internal antigens nucleoprotein (NP) and matrix protein 1 (M1) [5, 6], which are highly conserved with >90% homology among different strains [7]. M1 plays a pivotal role in influenza virus replication [8, 9]. Activation of these T-cell responses would mediate a broadly cross-reactive protection [10]. A number of novel T-cell–based influenza vaccines are being developed [11], including modified vaccinia Ankara virus (MVA)–vectored vaccines [12–14]. MVA-vectored vaccine expressing NP and M1 (MVA-NP+M1) is one of the promising vaccine candidates, showing activation of antigen-specific T-cell responses in peripheral blood after parenteral immunization [15, 16].

Tissue-resident memory T (T_RM_) cells reside in tissues and provide rapid response against reinfections at body surfaces [17]. These cells are anatomically positioned to quickly respond to local infection. Animal models showed that T_RM_ cells made critical contributions to protective immunity against local challenges and were much more effective than recirculating memory T cells [18–20]. A vaccine strategy that enables establishment and/or expands mucosal T_RM_ cells would have enormous potential for immediate protection against reinfection, offering more effective disease control [21].

Because influenza virus infects through nasopharyngeal mucosa, local intranasal vaccine delivery that activates cross-reactive mucosal T-cell immunity, including T_RM_ cells, offers an attractive strategy. Intranasal live attenuated influenza vaccine (LAIV) has been shown to induce local and systemic antibodies and T-cell immunity in children [22–24]. Aerosol delivery of a candidate universal influenza vaccine induced local cellular responses associated with partial protection against heterosubtypic influenza A in pigs [25]. Intranasal immunization relies on local nasopharynx-associated lymphoid tissue (NALT) to induce T- and B-cell responses. Adenotonsillar tissues are major components of human NALT and are known to be important induction sites for immunity against respiratory pathogens [26–28].

It was previously demonstrated that cross-reactive memory B-cell responses were primed after 2009 pdmH1N1 infection [29] and activation of NP-specific T-cell response by MVA-NP+M1 in human NALT [30]. Because M1 contains major immunodominant CD8^+^ T-cell epitopes and HLA-A2 is among the most common HLA alleles (20%–30%) [31], we examined HLA-A2 restricted M1_58–66_ peptide-specific CD8^+^ T-cell responses in adenotonsillar tissue after MVA-NP+M1 stimulation. We showed that MVA-NP+M1 elicits marked increases in M1-specific CD8^+^ T cells, including T_RM_ cells, which exhibit rapid degranulation and target cell killing on recall antigen recognition.

## METHODS

### Patients and Samples

Tonsillar tissues and peripheral blood samples were obtained from immunocompetent children and adults (aged 2–34 years) undergoing tonsillectomy owing to upper airway obstruction. Tissue samples were obtained from Alder Hey Children’s Hospital and Aintree University Hospital in Liverpool, United Kingdom. Demographic information for studied patients is summarized in Table 1. Patients who had any known immunodeficiency were excluded, as were those with grossly inflamed tonsillar tissues. Ethical approval was obtained (Reference no. 14/SS/1058), and informed consent was obtained in all cases.

**Table 1.** Study Patients by Age

Patient Group	Age, Mean (Range), y
Children	
Group 1 (n = 6)	2.5 (2–3.5)
Group 2 (n = 12)	5.7 (4–9)
Adults (n = 9)	20.6 (16–34)

### Vaccines and Peptides

MVA-NP+M1 is MVA virus expressing NP and M1 from A/Panama/2007/99, as a fusion protein joined by a 7–amino acid linker, from vaccinia p7.5 early/late promoter. Wild-type MVA was nonrecombinant MVA used as a vector control. Following the manufacturer’s instructions, 9-mer conserved peptides of influenza M1 (BEI resources) (Table 2) were reconstituted in 50% acetonitrile or dimethyl sulfoxide; 10 or 11 peptides were pooled at a concentration of 0.1 mg/mL per peptide. M1_58–66_ (GILGFVFTL) (IBA) was reconstituted in dimethyl sulfoxide (50%) at a final concentration of 1 mg/mL.

**Table 2.** 9-Mer Peptides of Conserved Major Histocompatibility Complex Class I Binding Epitopes from Matrix Protein 1 of Influenza A Viruses^a^

Peptide No. by Pool	Amino Acid Sequence	HLA Restriction
Pool 1		
1	29-EDVFAGKNT-37	HLA-A*03
2	31-VFAGKNTDL-39	HLA-A*2402, HLA-B*08
3	37-TDLEALMEW-45	HLA-A*01
4	49-RPILSPLTK-57	HLA-A*03
5	51-ILSPLTKGI-59	HLA-A*0201
6	56-TKGILGFVF-64	HLA-A*02
7	58-GILGFVFTL-66	HLA-A*02, HLA-A*2402
8	60-LGFVFTLTV-68	HLA-A*02
9	66-LTVPSERGL-74	HLA-A*02
10	68-VPSERGLQR-76	HLA-A*02
Pool 2		
11	71-ERGLQRRRF-79	HLA-A*02
12	75-QRRRFVQNA-83	HLA-A*02
13	76-RRRFVQNAL-84	HLA-A*02
14	122-GALASCMGL-130	HLA-B*35
15	123-ALASCMGLI-131	HLA-B*35
16	124-LASCMGLIY-132	HLA-B*35
17	126-SCMGLIYNR-134	HLA-B*35
18	177-NRMVLASTT-185	HLA-A*0301, HLA-A*11
19	179-MVLASTTAK-187	HLA-A*0301, HLA-A*11
20	180-VLASTTAKA-188	HLA-A*0301, HLA-A*11
21	181-LASTTAKAM-189	HLA-A*0301, HLA-A*11

^a^Obtained from BEI Resources (NR-2667).

### Fluorescence-Labeled Antibodies and M1 Tetramer

Fluorescence-labeled antibodies were used in flow cytometry, including antibodies to HLA-A2, CD19, CD3, CD11c, CD123, CD8, CD69, granzyme B, CD107a, interferon (IFN) γ, tumor necrosis factor (TNF) α, interleukin 2 (IL-2), CD20, CD38, CD27, immunoglobulin D (IgD), CCR7, CD45RA, and CD103 (BD Bioscience or Biolegend). Anti-M1 antibody (abcam) was conjugated with phycoerythrin (PE) using LYNX conjugation (Bio-Rad) to measure M1 expression. HLA-A02*01–GILGFVFTL (M1_58–66_)–PE tetramer (MBL) (M1-Tm) was used for staining M1-specific CD8^+^ T cells.

### Cell Isolation

Tonsillar mononuclear cells (MNCs) were isolated using density gradient centrifugation, as described elsewhere [32, 33]. Tonsillar MNCs were resuspended in Roswell Park Memorial Institute 1640 (RPMI 1640) medium containing HEPES, L-glutamine, 10% heat-inactivated fetal bovine serum, 100 U/mL penicillin and 100 μg/mL streptomycin (Gibco), termed “complete RPMI medium.” MNCs were screened for HLA-A2 type by means of flow cytometry.

### Measurement of M1 Expression in Tonsillar MNCs

Tonsillar MNCs were stimulated with MVA-NP+M1 at 1.0 multiplicity of infection and incubated for 18–20 hours. MNCs were stained for epithelial cell markers, including pancytokeratin and epithelial cellular adhesion molecule, and CD19/CD4/CD11c/CD123, followed by intracellular staining (Suppl figure S1) for M1 expression, using anti-M1 antibody. B-cell subsets were determined by means of fluorescence staining and identified as memory (CD19^+^CD20^+^CD38^−^CD27^+^IgD^−^), naive (CD19^+^CD20^+^CD38^−^IgD^+^CD27^−^) and germinal center B cells (CD19^+^CD20^+^CD38^+^) [34].

### Cell Stimulation for T-Cell Assays

Tonsillar MNCs were cocultured with either MVA-NP+M1 or wild-type MVA at 1 × 10^5^ plaque-forming units/mL. Cell culture in complete RPMI medium was supplemented with 2% autologous human plasma. Tonsillar MNCs were incubated for 7 days before any further experiments. Non–HLA-typed tonsillar MNCs were used for pooled-peptide stimulation and IFN-γ enzyme-linked immunospot (ELISPOT) assay, and MNCs from HLA-A2^+^ individuals were used to determine M1-specific CD8^+^ T-cell response with tetramer staining.

### IFN-γ ELISPOT Assay

At day 7 after culture, MVA-NP+M1-stimulated cells were rested in RPMI medium for 2 days, followed by IFN-γ ELISPOT assay (eBioscience). ELISPOT plates (Millipore) were coated with anti-IFN-γ antibody overnight. Next, 2 × 10^5^ cells stimulated with M1 peptide pools (10 μg/mL per peptide) were seeded in plate wells. Cells without stimulation were used as a negative control, and cells stimulated with SEB (BEI Resources) as a positive control. The plate was incubated for 24 hours, followed by addition of anti–IFN-γ detection antibody and avidin–horseradish peroxidase. Spots were developed by adding 3-amino-9-ethyl carbazole (Sigma) and counted using an EliSpot Reader.

### Detection of M1_58–66_-Specific CD8^+^ T Cells and T_RM_ Cells

For flow cytometric analysis of M1-Tm^+^ CD8^+^ T cells and their phenotypes in tonsillar tissue, freshly isolated tonsillar MNCs, or MNCs after coincubation with M1_58–66_ peptide for 2 days (to expand M1-Tm^+^ cells), were stained with HLA-A02*01-M1_58–66_-PE tetramer. HLA-A02*01 control tetramers including HLA-A02*01-HPV16 E7 (-YMLDLQPET) and HLA-A02*01–negative control tetramer (-ALAAAAAAV) (MBL) were used. The specific detection of M1-Tm^+^ cells in tonsillar MNCs was confirmed by positive staining in CD8^+^ T cells only by M1-Tm tetramer, and negative staining by control tetramers in MNCs after M1 peptide stimulation (data not shown). Tonsillar MNCs were also cocultured with MVA-NP+M1, followed by analysis of M1-Tm^+^ cells. For detection of M1-specific T_RM_, in addition to the above, MNCs were costained with anti-CD103, anti-CD69, anti-CD45RA, and anti-CCR7 antibodies.

### Measurement of T-Cell Proliferation

Tonsillar MNCs were labeled with carboxyfluorescein succinimidyl ester (CFSE; 5 μmol/L) (Invitrogen) [35]. CFSE-labeled cells were resuspended in RPMI medium supplemented with 2% autologous human plasma before stimulation with 1 × 10^5^ plaque-forming units/mL of MVA-NP+M1 for 5 days. Cells were then stained for CD8 and M1-Tm, followed by flow cytometry.

### Detection of CD107a Expression and Intracellular Cytokines

After 7-day MVA-NP+M1 stimulation, tonsillar MNCs were pulsed with 0.25 μg/mL M1_58–66_ peptide and cocultured with anti-CD107a antibody in the presence of brefeldin A and monensin (eBioscience). Cells were collected and stained for CD8 and M1-Tm and intracellular cytokines, followed by flow cytometry.

### Cytotoxic Killing Assay

Isolated CD8^+^ T cells after MVA-NP+M1 stimulation were cocultured with M1_58–66_-pulsed B cells, as described elsewhere [36]. Briefly, autologous B cells were isolated from cryopreserved tonsillar MNCs and incubated overnight with 40 ng/mL recombinant IFN-γ (Peprotech). B cells were then labeled with either 0.02 μmol/L (Target cell-CFSElow [T_low_]) or 0.2 μmol/L (Target cell-CFSEhigh [T_high_]) CFSE for 15 minutes. T_low_ cells were pulsed with 5 μg/mL M1_58–66_ for 45 minutes. Both T_low_ and T_high_ cells were adjusted to 2 × 10^5^ cells/mL and mixed at a 1:1 ratio. For effector cells, isolated CD8^+^ T cells after stimulation were adjusted to a cell count of 4–10 × 10^6^/mL before 2-fold serial dilutions were made (1:1 to 1:32). CD8^+^ T cells were then cocultured at different ratios with mixed T_low_ and T_high_ cells for 6 hours. Mixed T_low_ and T_high_ cells only (without CD8^+^ T cells) were cultured as negative control. Cells were harvested and stained with LIVE/DEAD Far Red stain (Invitrogen) for 30 minutes before staining for CD8 and M1-Tm.

### Flow Cytometry

Fluorescence-labeled cells were analyzed using a FACSCalibur cytometer with CellQuest v5.1 software or a FACSCelesta cytometer with FACSDiva v8.0.3 software (both BD) and analyzed using FlowJo 8.7 software.

### Statistical Analysis

For 2-group comparisons, based on normality of data, parametric paired *t* test, nonparametric Wilcoxon matched-pairs signed rank test and nonparametric Mann-Whitney test were performed using GraphPad Prism. Differences were considered statistically significant at *P* < .05.

## RESULTS

### M1 Antigen Expression in NALT After MVA-NP+M1 Stimulation

**Figure 1.**
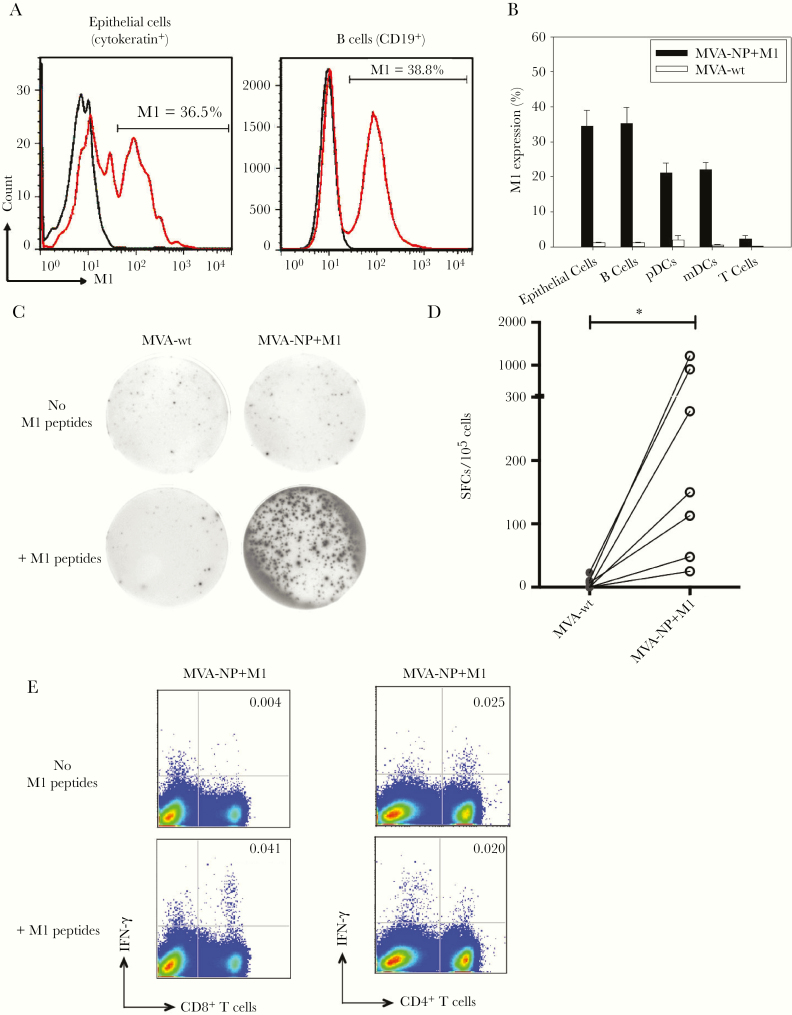
Expression of matrix protein 1 (M1) in tonsillar mononuclear cells (MNCs) after stimulation with modified vaccinia Ankara (MVA)–vectored vaccine expressing nucleoprotein (NP) and M1 (MVA-NP+M1), and T-cell responses to conserved M1 peptides. M1 expression was examined in tonsillar MNCs after either MVA-NP+M1 or wild-type MVA (MVA-wt) stimulation for 18 hours. *A,* Representative flow cytometric histograms showed the expression of M1 in tonsillar epithelial cells and B cells after stimulation by MVA-NP+M1 (*red line*) compared with MVA-wt (*black line*). *B,* Bar charts show the percentages of M1 expression in epithelial cells, B cells, plasmacytoid dendritic cells (pDCs), myeloid dendritic cells (mDCs), and T cells after MVA-NP+M1 stimulation, compared with MVA-wt stimulation (n = 3; values represent means with standard errors of the mean). C, After MVA-NP+M1 stimulation and cell resting, the frequency of interferon (IFN) γ–secreting T cells on restimulation by conserved M1 peptide pools were determined by means of IFN-γ enzyme-linked immunospot assay. Representative images showed spots (IFN-γ–secreting cells) in MNCs stimulated by MVA-NP+M1-versus MVA-wt, before and after restimulation by M1 peptide pools. *D,* IFN-γ spot-forming cell (SFC) counts in MNCs stimulated by MVA-NP+M1 or MVA-wt-stimulated MNCs followed by M1 peptide pool stimulation (n = 7). *P < .05, Wilcoxon signed rank test). SFC counts were obtained by subtracting background SFC count in cells without peptide restimulation. *E,* Representative dot plots showed a higher frequency of IFN-γ–producing CD8^+^ T cells than CD4^+^ T cells after restimulation by M1 peptide pools in MVA-NP+M1-stimulated MNCs (1 of 3 representative samples shown).

To determine whether M1 antigen was expressed in tonsillar cells after MVA-NP+M1 stimulation, we used intracellular M1 staining to examine M1 expression in tonsillar MNCs. As shown in Figure 1A and 1B, after stimulation, M1 was abundantly expressed in tonsillar epithelial cells (mean [standard error of the mean (SEM)], 34.5% [3.2%]) and B cells (35.2% [7.55%]), but only a small number of T cells (2.3% [0.6%]). Among B cells, M1 expression was detected in memory (mean [SEM], 55.8% [2.2%]), naive (48.7% [2.5%]), and germinal center (22.7 [0.9%]) B cells, respectively (data not shown). Among tonsillar dendritic cells (DCs), M1 expression was shown in myeloid DCs (mean [SEM], 21.2% [3.2%]) and plasmacytoid DCs (22.0% [7.1%]) (Figure 1B). As a control, no M1 expression was detected in any cell types after stimulation by MVA vector alone. MVA-NP+M1 elicited mucosal M1-specific T-cell responses.

Having shown abundant M1 expression in tonsillar MNCs, we investigated whether MVA-NP+M1 activated M1-specific T-cell responses. After MVA-NP+M1 stimulation, tonsillar MNCs were coincubated with 9-mer M1 peptide pools (Table 2), followed by IFN-γ ELISPOT assay. A marked increase in IFN-γ–secreting cells was found in MNCs stimulated by MVA-NP+M1, compared with those stimulated by MVA vector alone (Figure 1C and 1D; *P* < .05). Subsequent flow cytometry revealed that the increase in IFN-γ–secreting cells after M1 peptide restimulation was predominantly from CD8^+^ T cells and not from CD4^+^ T cells (Figure 1E), with a mean (SEM) increase of 0.27% (0.05%) of IFN-γ–secreting cells (percentage of CD8^+^ T cells). This suggests that MVA-NP+M1 stimulation activates a marked M1-specific T-cell response.

**Figure 2.**
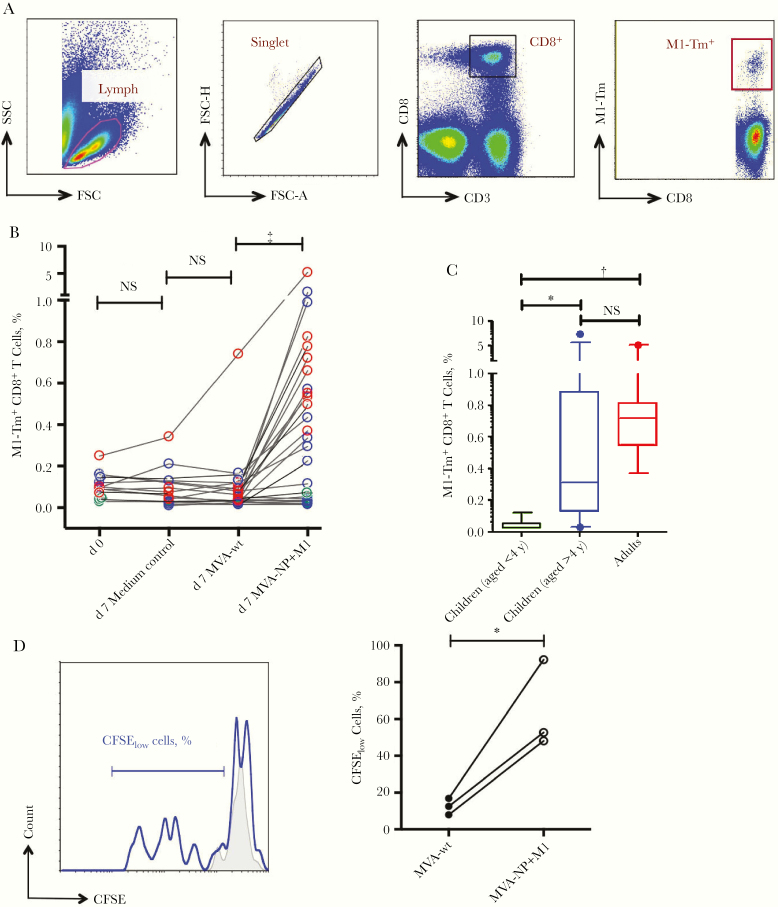
M1_58–66_ peptide-specific CD8^+^ T cells activated by modified vaccinia Ankara (MVA)–vectored vaccine expressing nucleoprotein (NP) and matrix protein 1 (M1) (MVA-NP+M1). M1_58–66_ peptide-specific CD8^+^ T cells (M1-Tm^+^) were identified using M1 tetramer staining in HLA-A2^+^ patients after 7-day culture of tonsillar mononuclear cells (MNCs) with MVA-NP+M1, wild-type MVA (MVA-wt), or medium control. *A,* Gating strategy for analysis of M1-Tm^+^ cells. *B,* MVA-NP+M1 activated an increase in M1-Tm^+^ compared with MVA-wt cells in children (green circle <4 years, blue >4 years) and adults (red circle) (n = 27). ‡*P* < .001; NS, not significant (Wilcoxon signed rank test). *C,* Frequency of M1-Tm^+^ cells among different age groups (displayed as medians with interquartile ranges). *P < .05; †*P* < .01. *D,* Gating on M1-Tm^+^ cells. Representative histogram shows that M1-Tm^+^ cell proliferation was activated by MVA-NP+M1 (*blue line*), compared with MVA-wt control (*gray shading*). *E,* Proliferation of M1-Tm^+^ cells (0.02 μmol/L [T_low_]) after stimulation of tonsillar MNCs by MVA-NP+M1, compared with MVA-wt control (n = 3). *P < .05 (Wilcoxon signed rank test).

To confirm this, we examined the M1-specific CD8^+^ T-cell response, using HLA-A2–restricted M1_58–66_-specific tetramer (Tm) staining in HLA-matched individuals (Figure 2A). Frequencies of M1-Tm^+^ cells in freshly isolated MNCs were generally low (median, 0.10%). MVA-NP+M1 stimulation elicited a marked increase in M1-Tm^+^ cells (median, 0.37%), compared with stimulation by MVA vector or medium control (Figure 2B; *P* < .001). When MVA-NP+M1-activated M1-Tm^+^ cell response was compared among different age groups (Table 1), an age-dependent increase was shown in M1-Tm^+^ cell response. In general, children <4 years old showed a low or modest response, and older children and adults demonstrated stronger responses (Figure 2C). Further analysis with CFSE cell tracing demonstrated that MVA-NP+M1 activated a proliferative M1-Tm^+^ cell response in tonsillar MNCs, compared with MVA vector only (Figure 2D; *P* < .05).

### M1-Specific T_RM_ Cell Response to MVA-NP+M1

To determine whether there were M1-specific T_RM_ cells in NALT and whether MVA-NP+M1 activated an increase in T_RM_ cells, we studied tonsillar MNCs from HLA-matched patients (aged 5–24 years) by costaining T_RM_ cell markers and M1 tetramer. Because frequencies of M1-Tm^+^ cells in ex vivo tonsillar tissue were low, we used M1-specific peptide to enrich M1-Tm^+^ cells in tonsillar MNCs (and in peripheral blood MNCs [PBMCs]) by coincubation with M1_58–66_ peptide for 2 days. 

**Figure 3.**
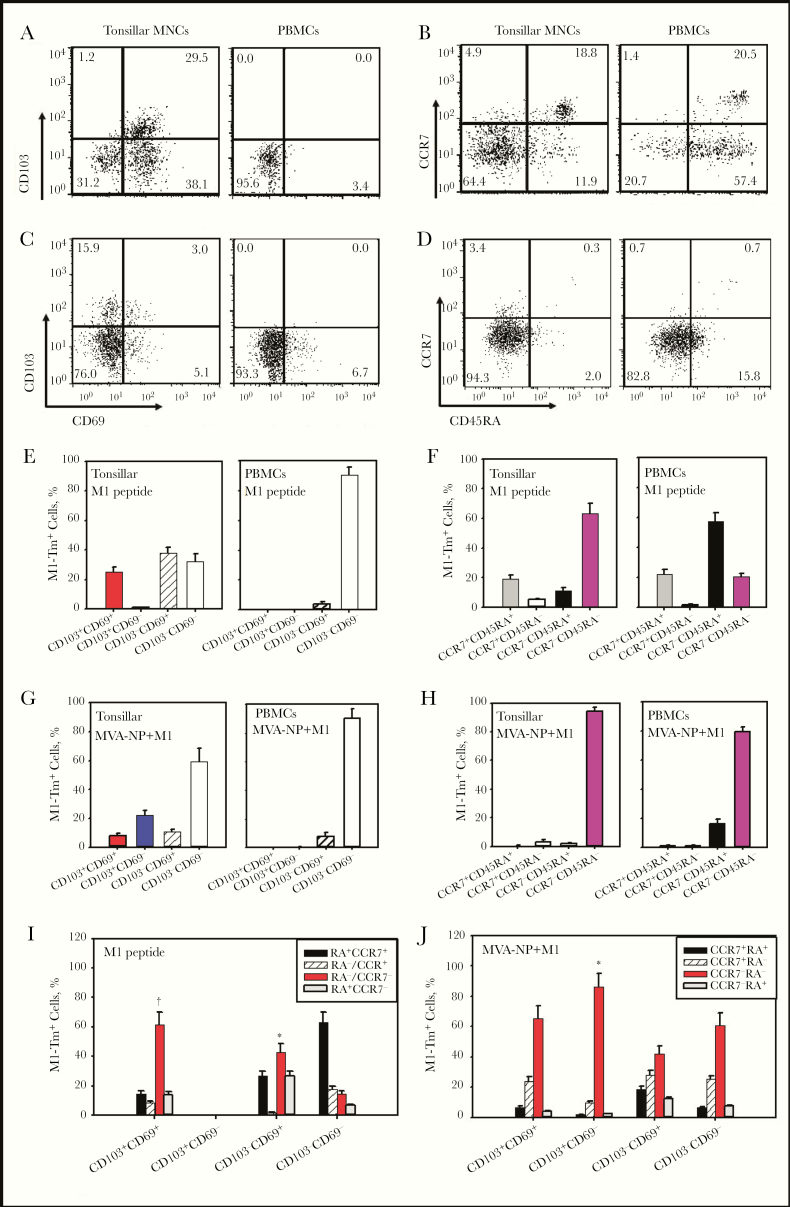
Matrix protein 1 (M1)–specific tissue-resident memory T (T_RM_)–cell response in tonsillar mononuclear cells (MNCs) activated by modified vaccinia Ankara (MVA)–vectored vaccine expressing nucleoprotein (NP) and M1 (MVA-NP+M1). *A, C,* Representative dot plots (gated on M1-Tm^+^ CD8^+^ T cells only) demonstrated the presence of preexisting M1_58–66_ peptide-specific T_RM_ cells (CD103^+^CD69^+^) in M1 peptide–expanded tonsillar MNCs and peripheral blood MNCs (PBMCs) (*A*) and substantially increased numbers of both CD103^+^ and CD103^−^ M1-Tm^+^ (M1-tetramer) cells after MVA-NP+M1 stimulation at day 7, particularly the increase in CD103^+^CD69^−^ subset in tonsillar MNCs (*C*). *A, C, E, G,* This contrasted with the findings in PBMCs showing the absence of CD103^+^CD69^+^ T_RM_ cells in both M1 peptide–expanded (*A, E*) and MVA-NP+M1-stimulated PBMCs (*C, G*). *B, D, F, H,* Memory phenotypes of M1-Tm^+^ cells were examined using CCR7 and CD45RA markers in tonsillar MNCs compared with PBMCs (M1 peptide–expanded [*B, F*] and MVA-NP+M1 stimulated [*D, H*]). *E–H,* T_RM_ and non-T_RM_ cell subsets (*E, G*) and their memory phenotypes (*F, H*) of M1-Tm^+^ cells in tonsillar MNCs and PBMCs after M1 peptide and MVA-NP+M1 stimulation. *I, J,* M1-Tm^+^ cell memory phenotypes in different T_RM_ and non-T_RM_ cell subsets in tonsillar MNCs after M1 peptide (*I*) or MVA-NP+M1 stimulation (*J*). *P < .05; †*P* < .01 (comparison with CD103^−^CD69^−^ non-T_RM_ cells; n = 5).

The phenotypes of expanded M1-Tm^+^ cells after peptide stimulation showed no difference in freshly isolated MNCs (data not shown). In tonsillar MNCs, a mean (SEM) of 25.1% (3.2%) M1-Tm^+^ cells expressed CD103^+^, therefore identified as M1-specific T_RM_ cells, and most of them were CD103^+^CD69^+^ T_RM_ cells (Figure 3A and 3E). There was also a mean (SEM) of 38.1% (3.6%) of M1-Tm^+^ cells expressing CD69 but not CD103 (CD103^−^CD69^+^). Of M1-Tm^+^ cells in MNCs, about 64% were of effector memory T-cell phenotype (CD45RA^−^CCR7^−^) (Figure 3B and 3F). Among M1-Tm^+^ cell subsets, the majority (mean [SEM], 64.2% [8.4%]) of CD103^+^CD69^+^ T_RM_ cells were of effector memory T-cell phenotype, compared with 42.6% (6.1%) and 14.4% (2.5%), respectively, for CD103^−^CD69^+^ and CD103^−^CD69^−^ subsets (Figure 3I). By contrast, in PBMCs from the same patients, no M1-Tm^+^ cells expressed CD103 (thus non-T_RM_ cells), and only approximately 20% were of CD45RA^−^CCR7^−^ effector memory phenotype, with the majority of CD45RA^+^CCR7^−^ phenotype (Figure 3B and 3F).

After MVA-NP+M1 stimulation, there was a substantial increase in M1-Tm^+^ cells (6–18-fold-increase), including both CD103^+^ and CD103^−^ cell subsets, and a large majority (approximately 90%) expressed CD45RA^−^CCR7^−^ phenotype (Figure 3C, 3G, and 3H). In CD103^+^ T_RM_ cells, there was a marked increase in CD103^+^CD69^−^ subset which accounted for approximately 75% of CD103^+^T_RM_ cells, whereas approximately 25% were CD103^+^CD69^+^ (Figure 3C and 3G). This contrasted with findings in freshly isolated MNCs or M1 peptide–expanded MNCs, in which CD103^+^ cells were primarily CD103^+^CD69^+^. Furthermore, when the memory phenotypes were analyzed, more CD103^+^CD69^−^ than CD103^+^CD69^+^ or CD103^−^CD69^+^ T_RM_ cells exhibited an effector memory phenotype (CD45RA^−^CCR7^−^) (mean, 86.1% vs 65.6% and 42.2%, respectively) (Figure 3J). When PBMCs from the same patients were analyzed, a marked increase in M1-Tm^+^ cells was also seen, but these cells in PBMCs were largely CD103^−^CD69^−^ non-T_RM_ cells (Figure 3C and 3G).

### Cytotoxic Functions and Killing Property of MVA-NP+M1-Activated M1-Specific CD8^+^ T Cells

**Figure 4.**
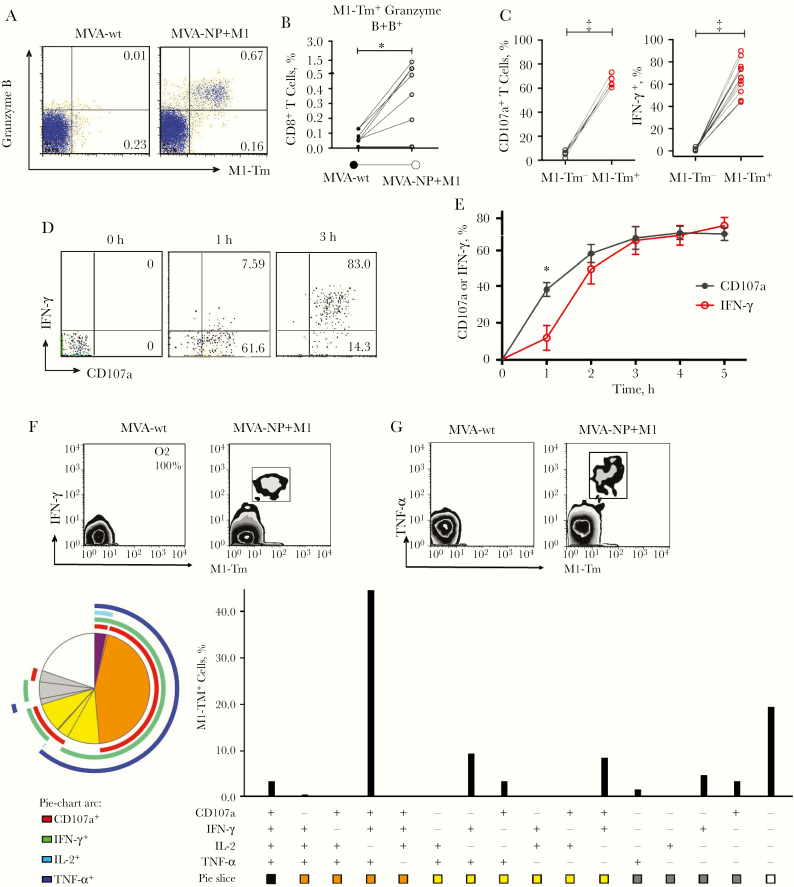
Cytotoxic molecule and proinflammatory cytokine expression profiles of matrix protein 1 (M1)–specific CD8^+^ T cells. Tonsillar mononuclear cells (MNCs) were stimulated by modified vaccinia Ankara (MVA)–vectored vaccine expressing nucleoprotein (NP) and M1 (MVA-NP+M1) for 7 days, followed by detection of M1-Tm^+^ (M1-tetramer) cells and expression of cytotoxic molecules. Tonsillar MNCs were subsequently pulsed with M1_58–66_ peptide for 6 hours, followed by detection of surface CD107a and intracellular cytokines. *A, B,* MVA-NP+M1-activated M1-Tm^+^ cells expressing high level of granzyme B, compared with wild-type MVA (MVA-wt) alone. *A,* Representative plots. *B,* Expression in 8 samples. *P < .05). *C,* After M1 peptide pulsing, both surface CD107a and intracellular interferon (IFN) γ were highly expressed in M1-Tm^+^ cells, compared with the low level in M1-Tm^−^ cells (n = 8 and n = 13 respectively). ‡*P* < .001). *D, E,* Representative dot plots (*D*) and kinetics curves (*E*) showed the coexpression of surface CD107a and intracellular IFN-γ in M1-Tm^+^ cells after peptide pulsing. At 1 hour, the percentages of CD107a^+^ cells were significantly higher than those of IFN-γ ^+^ cells (n = 4); mean and standard error of the mean are shown at each time point. *P < .05 (paired *t* test). *F, G,* Representative dot plots showed the high level of expression of IFN-γ (*F*) and tumor necrosis factor (TNF) α (*G*) in MVA-NP+M1-activated M1-Tm^+^ cells. *H,* Pie and bar charts demonstrate functional profile of M1-Tm^+^ cells in MVA-NP+M1-activated tonsillar MNCs followed by 6-hour restimulation with an M1_58–66_ peptide, showing coexpression of CD107a and 3 cytokines, IFN-γ, TNF-α, and interleukin 2 (IL-2) (1 of 2 representative samples is shown).

To determine whether MVA-NP+M1-activated M1-specific CD8^+^ T cells in tonsillar MNCs were functionally active, we examined the expression of cytotoxic molecules and cytokines in M1-Tm^+^ cells. At day 7 after vaccine stimulation, the M1-Tm^+^ cells expressed a high level of granzyme B (Figure 4A and 4B). Tonsillar MNCs were subsequently pulsed with M1_58–66_ peptide, followed by detection of surface CD107a (marker for degranulation) and cytokine expression. Both CD107a expression and IFN-γ expression were markedly up-regulated in M1-Tm^+^ cells after M1_58–66_ peptide pulsing (Figure 4C). The kinetics of CD107a and IFN-γ expression were further studied and a similar pattern was shown for both (Figure 4D and 4E). Notably, the up-regulation in expression was more rapid in CD107a than in IFN-γ. 

At 1 hour after peptide pulsing, approximately 40% of M1-Tm^+^ cells expressed CD107a, compared with 10% producing IFN-γ (*P* < .05). Both surface CD107a expression and IFN-γ production seemed to peak after 3 hours (Figure 4D and 4E). IFN-γ and TNF-α were abundantly expressed in M1-Tm^+^ cells after peptide pulsing (Figure 4F and 4G). Figure 4H shows the frequencies of M1-Tm^+^ cells expressing different cytokine profiles, with the most frequently detected M1-Tm^+^ cells coexpressing CD107a with IFN-γ and TNF-α (45%). Some M1-Tm^+^ cells (3%) were shown to coexpress CD107a and 3 cytokines, IFN-γ, TNF-α, and IL-2 (Figure 4H).

**Figure 5.**
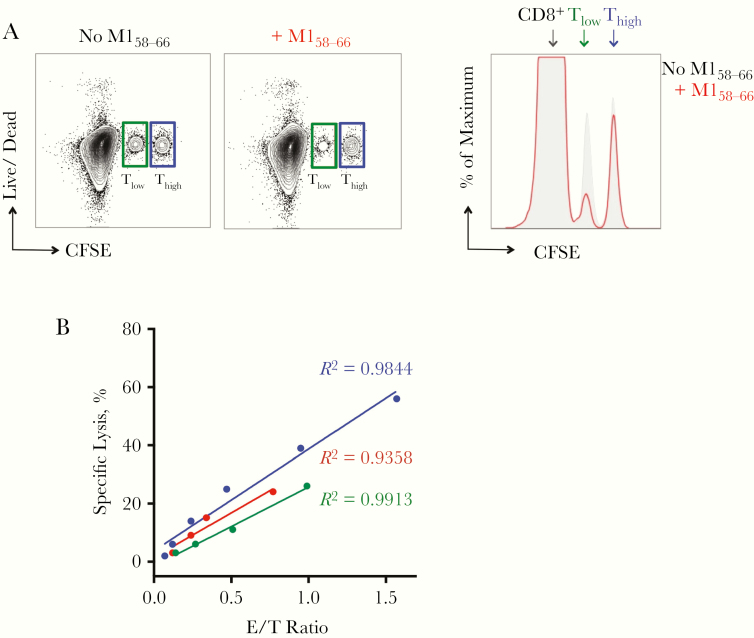
Specific killing capacity of M1_58–66_ peptide-specific CD8^+^ T cells. Isolated CD8^+^ T cells after stimulation by modified vaccinia Ankara (MVA)–vectored vaccine expressing nucleoprotein (NP) and matrix protein 1 (M1) (MVA-NP+M1) were cocultured at different ratios with autologous B cells labeled with low (T_low_) and high (T_high_) with carboxyfluorescein succinimidyl ester (CFSE) intensities. T_low_ cells were either pulsed with M1_58–66_ peptide or were without pulsing, and T_high_ cells were without pulsing. *A,* Representative dot plots and histogram demonstrating the decrease in target cells (T_low_) after M1 peptide pulsing (*green gate or middle peak*), compared with nonpulsing controls (*gray shading*), indicating M1-specific target cell killing_._*B,* Correlations between percentages of M1-specific target cell lysis and effector-target (E/T) cell ratio in 3 patients. *E* refers to effector number of M1-Tm^+^ (M1-tetramer) cells of isolated CD8^+^ T cells, and *T* to number of target T_low_ cells. The proportion of M1-Tm^+^ cells in the total isolated CD8^+^ T cells ranged from 1% to 4%.

We further investigated whether M1-Tm^+^ cells were capable of the cytotoxic killing of target cells. After MVA-NP+M1 stimulation, isolated CD8^+^ T cells (as effector T cells) were cocultured with M1_58–66_ peptide-pulsed target B cells, followed by flow cytometric measurement of target cell lysis. As demonstrated in Figure 5A, there was a marked decrease in peptide-pulsed target B cells (T_low_) but no decrease in B cells without peptide-pulsing (T_high_) after coculture with effector T cells, indicating M1-specific target cell lysis. In all 3 samples tested, the increase in target cell lysis was correlated well with the increase in effector-target cell ratio (Figure 5B).

## DISCUSSION

Because intranasal vaccination is considered an effective vaccination strategy against respiratory pathogens [22–24], we investigated the potential of MVA-NP+M1 as a mucosal vaccine to activate anti-influenza T-cell responses in human NALT. We demonstrated that MVA-NP+M1 activates a prominent M1-specific cytotoxic T-cell response with a marked increase in M1-specific T_RM_ cells.

After MVA-NP+M1 stimulation, we showed that M1 antigen was highly expressed in both tonsillar epithelial cells and B cells. This suggest that MVA-NP+M1 has the capacity to efficiently infect tonsillar cryptal epithelium and present M1 antigen. Tonsillar tissue has a reticular crypt epithelium containing both epithelial and nonepithelial immune cells. Efficient infection of epithelium by MVA-vectored vaccine would provide a favorable environment for vaccine uptake and antigen presentation. Memory B cells, representing a major nonepithelial immune cell subset, were found mainly within intraepithelial areas and have a strong capacity to present antigen directly to T cells, owing to the constitutive expression of costimulatory molecules [37–39]. 

The unique anatomic localization of memory B cells in intraepithelial areas, together with the strong antigen-presenting capacity, has been considered critical for prompt and robust memory antibody responses [37]. It is therefore possible that memory B cells are infected by the MVA vaccine virus and efficiently present the vaccine antigen (eg, M1) to memory T cells, contributing to activation of memory T cells in tonsillar MNCs. DCs may also contribute to vaccine uptake and antigen processing, because significant proportions of myeloid and plasmacytoid DCs showed M1 expression, consistent with previous findings [40].

With IFN-γ ELISPOT assay, we demonstrated that MVA-NP+M1 activated a marked increase in IFN-γ–secreting CD8^+^ T cells specific to conserved M1 epitopes. Furthermore, using M1_58–66_-specific tetramer staining, we showed that MVA-NP+M1 stimulation elicited a marked increase in M1-Tm^+^ T cells in tonsillar MNCs from HLA-matched individuals, particularly in older children and adults. M1_58–66_-specific CD8^+^ T cells have been shown to protect against influenza infection in HLA-A2 transgenic mice [41]. Our results therefore provide evidence supporting the capacity of MVA-NP+M1 to elicit M1-specific CD8^+^ T-cell responses with the potential for protection against influenza in the human nasopharynx.

Recent research supports a critical role of T_RM_ cells in providing a rapid protection against influenza. T_RM_ cells in human lungs were shown to mount a rapid response and kill influenza-infected epithelial cells and contribute to protection [42, 43]. Using M1 tetramer and CD103/CD69 costaining, we demonstrated the presence of CD103^+^ M1-specific CD8^+^ T_RM_ cells in tonsillar tissue, which were expanded by M1-specific peptide. M1-Tm^+^ cells included were both CD103^+^CD69^+^ and CD103^−^CD69^+^ T_RM_ subsets. Similar to findings of a previous study on EBV-specific T_RM_ cells in tonsillar tissue [44], M1-specific CD103^+^ cells were largely restricted to CD69^+^ cells, and a large proportion of these T_RM_ cells were of effector memory T-cell phenotype. It has been shown that CD103^+^CD69^+^ T_RM_ cells preferentially localized to tonsillar epithelial surface, whereas CD103^−^CD69^+^ cells largely localized in extrafolicular regions [44]. Our results therefore support the presence of M1-specific T_RM_ cells in tonsillar epithelium, derived from memory T cells primed by previous influenza infection. These cells largely exhibit effector memory T-cell phenotype, with the ability to mount a fast response to reinfection.

After MNA-NP+M1 stimulation, there was an increase in M1-specific T_RM_ cells (CD103^+^) and non-T_RM_ cells (CD103^−^) in tonsillar MNCs. Interestingly, of CD103^+^ T_RM_ cells, the majority were CD103^+^CD69^−^, whereas only approximately 25% were CD103^+^CD69^+^, cells which were predominant in unstimulated tonsillar MNCs. It would be interesting to know whether there is any functional difference between CD103^+^CD69^−^ and CD103^+^CD69^+^ subsets in future studies. The fact that a large majority of CD103^+^CD69^−^ cells exhibited effector memory T-cell phenotype indicates they have the capacity to respond to reinfection rapidly. 

These results suggest that MVA-NP+M1, if used as an intranasal vaccine, could elicit a proliferative response of T_RM_ cells, to expand the T_RM_ memory T-cell pool in NALT and offer rapid protection against influenza infection in the nasopharynx. MVA-NP+M1 most likely acts by boosting preexisting memory CD8^+^ T cells, but not by inducing de novo M1-specific T cells, because tonsillar MNCs depleted of memory T cells (CD45RO^+^) failed to show any M1-Tm^+^ cells after MVA-NP+M1 stimulation (data not shown).As a comparison, we also analyzed M1-Tm^+^ T cells in PBMCs, and we demonstrated the absence of T_RM_ (CD103^+^CD69^+^) cells in PBMCs before and after the vaccine stimulation. This supports the concept that CD103^+^ T_RM_ cells are retained in peripheral tissue but not present in the circulation. Local mucosal vaccination may therefore offer distinctive advantage in expanding antigen-specific T_RM_ cells in local tissues for rapid protection.

It is generally thought that cytotoxic CD8^+^ T cells exert their effector activities to limit virus infection and disease severity [6, 10] through degranulation, cytotoxic molecule release, and proinflammatory cytokines [45]. In the current study, we demonstrated that M1-Tm^+^ cells activated by MVA-NP+M1 expressed a high level of granzyme B, which was subsequently released on recognition of M1_58–66_ peptide, along with rapid up-regulation of surface CD107a expression. In addition, many M1-Tm^+^ cells coexpressed CD107a with IFN-γ and TNF-α, suggesting that they produce both cytotoxic effector molecules and inflammatory cytokines on antigen-specific recognition. IFN-γ and TNF-α are potent proinflammatory cytokines and important in antiviral activity. In addition to these 2 cytokines, some of these cells also coexpressed IL-2, which may exhibit more potent cytotoxic functions [46, 47]. Although CD4^+^ cells, rather than CD8^+^ T cells, are the main source of IL-2, a small number of CD8^+^ T cells can secrete IL-2 after receiving costimulatory signals, providing proliferation and survival signals to themselves or other cytotoxic T cells [45].

The kinetics of CD107a expression correlated well with that of cytokine (IFN-γ) production in the M1-Tm^+^ cells. The rapid up-regulation of surface CD107a expression (ie, degranulation) in M1-Tm^+^ cells on specific antigen recognition suggests that these M1-specific CD8^+^ T cells, including T_RM_ cells, may mount an immediate cytotoxic response against influenza. Finally, using M1-specific peptide pulsed tonsillar B lymphocytes as target cells for the effector T-cell function, we showed thatMVA-NP+M1-activated M1-Tm^+^ cells had marked cytotoxic killing activity capable of target cell lysis.

In conclusion, we demonstrate that MVA-NP+M1 activated an M1-specific mucosal CD8^+^ T-cell response, including a substantial increase in T_RM_ cells. These M1-specific T cells were predominantly of effector memory T-cell phenotype, exhibiting a high level of cytotoxic markers and producing proinflammatory cytokines, leading to specific killing of target cells on antigen recognition. Our results suggest that this novel vaccine expands the M1-specific T_RM_ cell pool and activates cytotoxic T-cell responses to the conserved antigen, therefore offering great potential as an effective mucosal vaccine for fast and broad protection against reinfection of influenza virus in humans.

## Supplementary Data

Supplementary materials are available at *The Journal of Infectious Diseases* online. Consisting of data provided by the authors to benefit the reader, the posted materials are not copyedited and are the sole responsibility of the authors, so questions or comments should be addressed to the corresponding author.

jiz593_suppl_Supplemental_Figure_1

## Notes

***Acknowledgments.*** We thank the patients who took part in the study and the operating theatre staff at Liverpool Children’s Hospital and Aintree University Hospital for helping collect samples. The reagent (NR-2667) was obtained through BEI Resources, National Institute of Allergy and Infectious Diseases, National Institutes of Health.

***Author contributions.*** S. P., M. S. A., P. S. M., S. C. G., and Q. Z. conceived and designed the experiments. S. P., M. S. A., and Q. Z. performed the experiments and analysis. R. S., M. K., S. L., T. L., and S. C. G. contributed clinical samples and vaccines/materials. All authors helped write the manuscript.

***Disclaimer.*** The views expressed are those of the authors and not necessarily those of the funders, the National Health Service, or the UK Department of Health. The funders had no role in study design, data collection and interpretation, or the decision to submit the work for publication.

***Financial support.*** This work was supported by SPARKS Medical Research (Q. Z.), United Kingdom (grant 15Liv01), the British Medical Association (H. C. Roscoe award [Q. Z.]), the Government Pharmaceutical Organization, Thailand (studentship [S. P.]), and the National Institute for Health Research Alder Hey Clinical Research Facility.

***Potential conflicts of interest.*** S. C. G. is an inventor on patents covering MVA-NP+M1, filed and owned by the University of Oxford, and is a cofounder of and consultant to Vaccitech, a University of Oxford spin-off company undertaking advanced clinical development of viral vectored influenza vaccines. All other authors report no potential conflicts. The authors have submitted the ICMJE Form for Disclosure of Potential Conflicts of Interest. Conflicts that the editors consider relevant to the content of the manuscript have been disclosed.
