# Supplementary figures and images for: Modified Vaccinia Ankara–Vectored Vaccine Expressing Nucleoprotein and Matrix Protein 1 (M1) Activates Mucosal M1-Specific T-Cell Immunity and Tissue-Resident Memory T Cells in Human Nasopharynx-Associated Lymphoid Tissue
Source: J Infect Dis. 2019 Nov 19;222(5):807–19. doi: 10.1093/infdis/jiz593 (PMC7399703; doi:10.1093/infdis/jiz593)

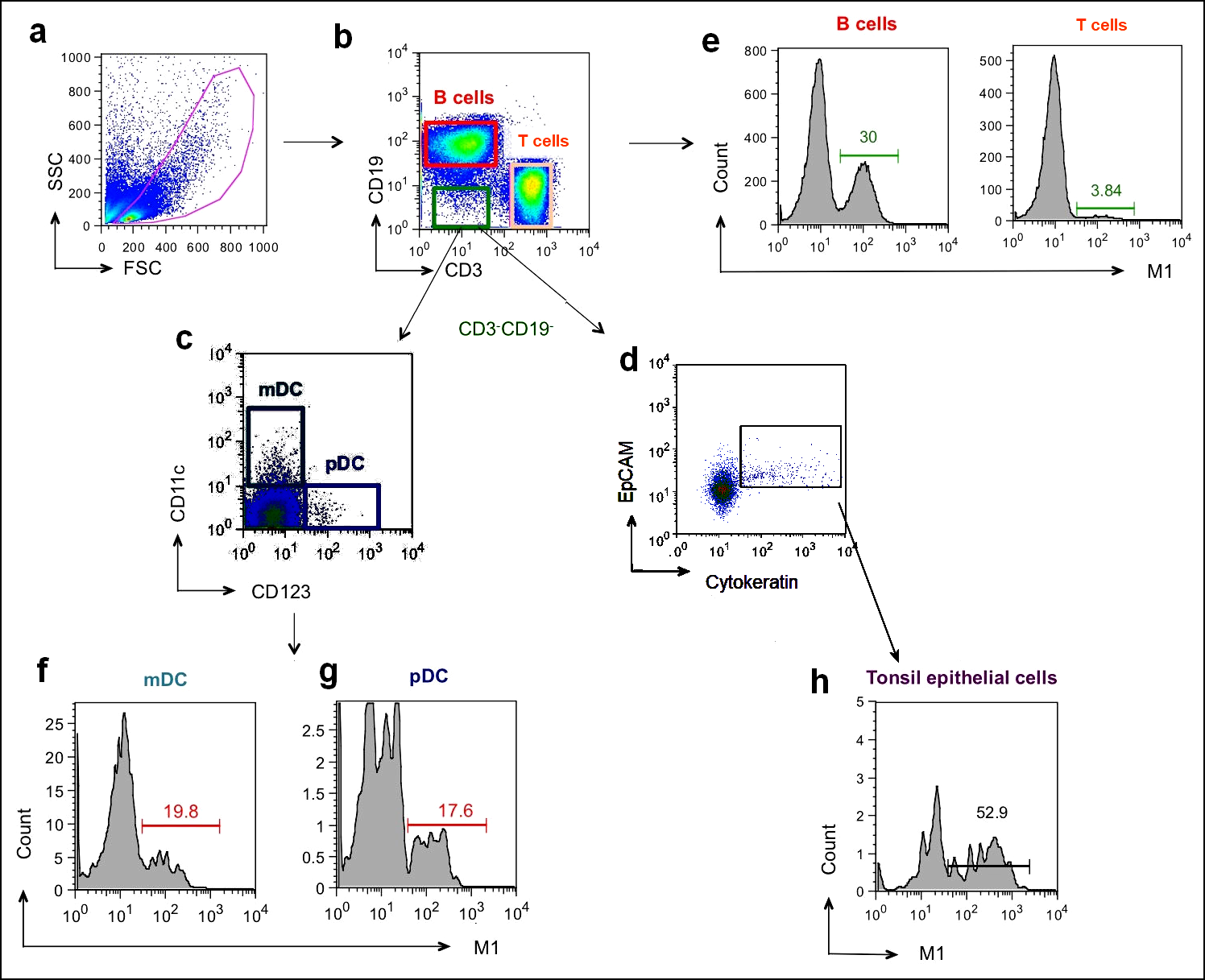

Supplement: jiz593_suppl_Supplemental_Figure_1 [file jiz593_suppl_supplemental_figure_1.png]
